# Vitamin D3 Suppresses Human Cytomegalovirus-Induced Vascular Endothelial Apoptosis *via* Rectification of Paradoxical m6A Modification of Mitochondrial Calcium Uniporter mRNA, Which Is Regulated by METTL3 and YTHDF3

**DOI:** 10.3389/fmicb.2022.861734

**Published:** 2022-03-11

**Authors:** Wenbo Zhu, Hongbo Zhang, Shao Wang

**Affiliations:** ^1^Clinical Medical Research Center, First Affiliated Hospital, University of South China, Hengyang, China; ^2^Department of Microbiology and Immunology, LSU Health Sciences Center, Shreveport, LA, United States; ^3^Institute of Animal Husbandry and Veterinary Medicine, Fujian Academy of Agriculture Science, Fuzhou, China

**Keywords:** HCMV, vascular endothelial cells, apoptosis, MCU, m6A modification

## Abstract

Human cytomegalovirus (HCMV) infection can induce apoptosis of vascular endothelial cells, which may be the most important element of development and progression of reported atherosclerosis caused by HCMV. As there are no specific drugs to clear HCMV infection, exploration of relevant drugs and mechanisms that can intervene in HCMV-induced atherosclerosis is urgently needed. The present study confirmed that vitamin D3 protected vascular endothelial cells from HCMV-induced apoptosis by inhibiting endoplasmic reticulum (ER) and mitochondrial apoptosis pathway. Mechanistically, HCMV infection could induce aberrantly elevated m6A modification, especially the increases of methyltransferases-“writers” (METTL3) and m^6^A binding proteins-“readers” (YTHDF3). METTL3 methylates mitochondrial calcium uniporter (MCU), the main contributor to HCMV-induced apoptosis of vascular endothelial cells, at three m6A residues in the 3′-UTR, which promotes the association of the YTHDF3 with methylated MCU mRNA and subsequently increases the translation and expression of MCU. Further analysis shows that ALKBH5 is the demethylases-“eraser” of MCU mRNA, which can negatively regulate the m6A modification process of MCU. Conversely, vitamin D3 downregulated the METTL3 by inhibiting the activation of AMPK, thereby inhibiting the m6A modification of MCU and cell apoptosis. Our findings extend the understanding of m6A driven machinery in virus-induced vascular endothelium damage and highlight the significance of vitamin D3 in the intervention of HCMV-induced atherosclerosis.

## Introduction

Human cytomegalovirus (HCMV) is a member of the *Herpesviridae* family and infects a broad range of cell types in human beings. In the normal human population, it can establish a latent infection with a mostly asymptomatic clinical outcome. However, reactivation from latent state under some circumstances or infection of an immunocompromised population can lead to severe clinical symptoms and even lethal outcomes (Khoshnevis and Tyring, 2002; Crough and Khanna, 2009; Manandhar et al., 2019). Apoptosis is a programmed cell death process that can be regulated by a variety of signals. On the one hand, HCMV viruses have developed a set of apoptosis-inhibiting mechanisms in order to establish a long-term persistent infection (Zhu et al., 1995; Goldmacher, 2005; Andoniou and Degli-Esposti, 2006; Brune, 2011). On the other hand, HCMV can also induce apoptosis in many cell types, mediating HCMV-induced pathological processes and viral transmission (Moon et al., 2003; Lee et al., 2013; Nakamura et al., 2013; Shao et al., 2016). It is unclear when HCMV induces apoptosis and when it causes apoptosis inhibition, probably in the context of different cellular environments and virus strains. Notably, HCMV infection can induce apoptosis of vascular endothelial cells (Shen et al., 2004; Utama et al., 2006), which may be closely related to the reported atherosclerosis caused by HCMV.

Vitamin D is a multifunctional lipid soluble hormone that is essential in a variety of physiological and pathological processes. Vitamin D3 and its metabolites regulate the transcription of target genes and exert biological effects through the vitamin D receptor (VDR). Accumulating evidence has shown that vitamin D3 deficiency is strongly associated with the development of atherosclerosis (Mozos and Marginean, 2015; Bennett and Lavie, 2017; Chen et al., 2018). Multiple mechanisms involved in occurrence and development of atherosclerosis are regulated by the VDR signaling, including improving endothelial function (Kim et al., 2020), inhibiting the formation of foam cells (Oh et al., 2009; Szeto et al., 2012), and suppressing the proliferation of vascular smooth muscle cells (Chen et al., 2010). Mostly, vitamin D3 can protect endothelial cells from apoptosis induced by radiation, immune damage, and oxidative stress (Dehghani et al., 2013; Polidoro et al., 2013; Marampon et al., 2016). However, whether vitamin D3 can protect vascular endothelial cells from HCMV-induced apoptosis is not yet known. As there are no specific drugs to clear HCMV infection, exploring related mechanisms can help us find interventions for HCMV-induced atherosclerosis.

N^6^-methyladenosine (m^6^A), the most prevalent post-transcriptional modification (PTMs) in mammalian mRNAs, is involved in a variety of physiological and pathological processes (Song et al., 2019). The m^6^A modification can be dynamically regulated by methyltransferases-“writers” (METTL3, METTL14, and WTAP), demethylases-“erasers” (ALKBH5 and FTO), and m^6^A binding proteins-“readers” (YTHDF1-3 and YTHDC1). This epigenetic regulatory mechanism has been reported to modulate alternative splicing, RNA stability, translation efficiency, and nuclear export (Wang et al., 2014, 2015; Zhao et al., 2014; Xiao et al., 2016). However, the status of m6A modification and the underlying regulatory mechanisms in HCMV-induced pathology are not fully understood. Mitochondrial calcium uniporter (MCU) located on the mitochondrial inner membrane is an important channel protein mediating mitochondrial calcium uptake. A lot of evidence has shown that MCU can promote apoptosis through the mitochondrial pathway and endoplasmic reticulum stress (Guan et al., 2019; Li et al., 2020). The previous study indicated that PTMs such as AMP-activated protein kinase-dependent phosphorylation regulated the mitochondrial calcium uptake and activity of MCU (Zhao et al., 2019). However, it is still unknown whether m^6^A modification at the nucleotide level can control the activity and expression of MCU and which enzyme mediates this modification. Understanding these issues will provide the opportunity to develop new therapeutic strategies to control the occurrence of apoptosis.

In the present study, utilizing an HCMV VHL/E-infected human aortic endothelial cell apoptosis model, we found for the first time that vitamin D3 protects vascular endothelial cells from HCMV-induced apoptosis by reducing the elevated translation of MCU induced by HCMV through METTL3- and YTHDF3-dependent mechanisms *via* VDR/AMPK/METTL3 pathway.

## Materials and Methods

### Cell Lines and Virus Infection

Primary human aortic endothelial cells (HAECs) were obtained from PromoCell (Heidelberg, Germany) and cultured or passaged in the endothelial cell growth medium MV2 (PromoCell, C-22022, Germany) supplemented with 10% fetal bovine serum and 1% penicillin–streptomycin at 37°C in a humidified atmosphere of 5% CO_2_. Cell detachment was performed with 0.05% trypsin in ethylene diamine tetra acetic acid (EDTA; Thermo Fisher, 25300120, United States). All cells used were between passages 3 and 8.

The human cytomegalovirus (HCMV) VHL/E strain was used in this study. The virus was propagated in Retinal Pigment epithelial cells (RPE-1). The RPE-1 cells were harvested after HCMV infection and underwent a freeze /thaw process to release the virus. Infection of HAECs was conducted at a multiplicity of infection (MOI) of 5 as determined by titration on RPE-1 cells. Then, the HAECs were incubated at 37°C for 2 h to absorb the virus, washed three times with phosphate buffer solution (PBS), and added with fresh complete medium. Cell fractions or supernatants were harvested at various postinfectious times.

### Chemical Reagents and Antibodies

Chemical reagents used in this study and indicated working concentrations were as follows: 1α,25(OH)2-vitamin D3 (100nM, 10 nM, and 1 nM; Sigma, United States, D1530), AICAR (1 mM; Sigma, United States, A9978), Spermine (10 μM; Sigma, United States, S3256), and Ru360 (5 μM; Sigma, United States, 557440). The following antibodies were used as: anti-p53 (Cell Signaling, #9282, United States), anti-Bax (Cell Signaling, #5023, United States), anti-Bcl-2 (Cell Signaling, #2870, United States), anti-cytochrome *c* (Cell Signaling, #4272, United States), anti-CHOP (Abcam, ab11419, United States), anti-GRP78 (Abcam, ab21685, United States), anti-beta actin (Proteintech, 66009-1-Ig, United States), anti-m6A antibody (Invitrogen, MA5-33030, United States), anti-METTL3 (Abcam, ab195352, United States), anti-METTL14 (Abcam, ab220030, United States), anti-YTHDC1 (Abcam, ab259990, United States), anti-YTHDF1 (Abcam, ab220162, United States), anti-YTHDF2 (Abcam, ab220163, United States), anti-YTHDF3 (Abcam, ab255267, United States), anti-ALKBH5 (Abcam, ab195377, United States), anti-FTO (Abcam, ab126605, United States), anti-phospho-AMPK alpha2 (Abcam, ab109402, United States), anti-AMPK alpha 2 (Abcam, ab214425, United States), anti-VDR (Proteintech, 67192-1-Ig, United States), anti-MCU (Abcam, ab219827, United States), and anti-Cytomegalovirus glycoprotein B antibody (Abcam, ab6499, United States).

### Cell Transfection, Plasmids, and siRNA Knockdown

MCU-3′UTR with either wild type or mutant (m6A site was replaced by T) were inserted into downstream of pGL3 Luciferase Reporter Vectors (Promega, E1751, United States) to construct pGL3-MCU-WT plasmid or pGL3-MCU-mut plasmid. The full-length and truncated forms of the human gene [YTHDF1 (GenBank accession no. NP_060268.2), YTHDF2 (GenBank accession no. NP_001166599.1), YTHDF3 (GenBank accession no. NP_001264742.1), △YTH, and YTH], each with a hemagglutinin (HA) tag sequence (YPYDVPDYA), were amplified by PCR and cloned into HindIII and BamHI sites of pcDNA3.1(+) (Invitrogen, V87020, United States) to produce pHA-YTHDF1, pHA-YTHDF2, pHA-YTHDF3, pHA-△YTH, and pHA-YTH. Human METTL3 (GenBank accession no. NP_062826.2) or human ALKBH5 (GenBank accession no. NP_060228.3) or human FTO (GenBank accession no. NP_001073901.1) cDNA was generated by PCR and cloned into pFlag-CMV2 expression plasmid (Sigma, E7033, United States) to produce pFlag-METTL3, pFlag-ALKBH5, or pFlag-FTO.

All RNA duplexes were obtained from GenePharma Company (Shanghai, China). The corresponding sequences are listed as follows:

**Table 1:** 

Gene	siRNAs sequences
METTL3	siMETTL3-1: 5’-CUGCAAGUAUGUUCACUAUGA-3’
	siMETTL3-2: 5’-AGGAGCCAGCCAAGAAAUCAA-3’
METTL14	siMETTL14: 5’-UUUAACACGGCACCAAUGC-3’
ALKBH5	siALKBH5-1: 5’-ACAAGUACUUCUUCGGCGA-3’
	siALKBH5-2: 5’-GCGCCGUCAUCAACGACUA-3’
FTO	siFTO: 5’-AAAUAGCCGCUGCUUGUGAGA-3’
YTHDF1	siYTHDF1: 5’-UUCAUGAACAACUAGACGCGG-3’
YTHDF2	siYTHDF2: 5’-GCCCAAUAAUGCAUAUACU-3’
YTHDF3	siYTHDF3-1: 5’-UUCGCCUGUUUCCUCUGCU-3’
	siYTHDF3-2: 5’-UAUCAUUGCCAAAUCCAGC-3’
MCU	siMCU: 5’-GCCAGAGACAGACAAUACU-3’
VDR	siVDR: 5’-AGUUCAUUCUGACAGAUGA-3’
Negative control	5’-UUCUCCGAACGUGUCACGU-3’

Cell transfection was performed by using Lipofectamine 2000 (Invitrogen; Thermo Fisher Scientific, 11668019, United States) for plasmids and Lipofectamine RNAiMAX (Invitrogen, Thermo Fisher Scientific, 13778030, United States) for siRNA according to the manufacturer’s protocols.

### Luciferase Reporter Assay

Primary human aortic endothelial cells were plated at a density of 2 × 10^5^ per well in a 12-well plate. HAECs in each were transiently transfected with 750 ng pGL3 Luciferase Reporter Vectors (Promega, E1751, United States) fused with or without the wild-type or mutated MCU-3′-UTR and 100 ng Renilla luciferase reporter vectors plus METTL3 siRNAs or YTHDF1-3 siRNAs using Lipofectamine 3000 (Thermo Fisher Scientific, L3000015, United States). At 12 h post-transfection, cells were infected with the indicated dose of HCMV. Cells were harvested 24 h after infection, and a dual-luciferase reporter assay kit (Promega, E1910, United States) was applied to assess the luciferase activities according to the manufacturer’s instructions. Renilla luciferase activity was used to standardize transfection efficiency. Each experiment was subjected to at least three times replication.

### Western Blot Assay

Indicated cells were lysed using high intensive radio immunoprecipitation assay (RIPA) buffer (Beyotime, China, P0013B). The cell suspension was centrifuged at 14,000 × *g* for 10 min at 4°C. The protein supernatant was collected and concentration was determined by a BCA Assay Kit (CWBIO, China, CW0014S). The equal amounts of protein were separated by sodium dodecyl sulfate-polyacrylamide gel electrophoresis (SDS-PAGE) at 100 V for 1.5 h and electrotransfer into polyvinylidene difluoride membranes (PVDF; Millipore, ISEQ00010, United States) at 200 mA for 2 h. Then, the membranes were blocked with 5% non-fat milk for 1 h at room temperature and incubated with the indicated primary antibodies overnight at 4°C. Next day, after being washed three times with TBST [20 mM Tris–HCl, 500 mM NaCl, and 0.1% Tween 20 (pH 7.5)], membranes were incubated with HRP-conjugated secondary antibodies at indicated concentration for 2 h at room temperature. The signals were detected using Super-enhanced chemiluminescence detection reagents (Applygen Technologies, P1030, China). The intensity of the protein bands was quantified using NIH ImageJ software.

To detect phosphorylated MCU, phosphorylation-tag gels (Jingke, China, 195-17991) containing Zn^2+^ ions were used for SDS-PAGE, which can specifically capture phosphorylated proteins in gel. After Western blotting with anti-MCU antibodies, phosphorylated proteins were showed as slower migration bands compared to corresponding unphosphorylated proteins.

### Caspase-3/9 Activity Assay

Caspase-3 and caspase-9 activities were measured using 30 μg protein obtained from extracted cells and Caspase-3/9 Activity Assay Kit (Beyotime Institute of Biotechnology; China; catalog no. C1115 and C1158) following the manufacturer’s instructions. Cells were incubated with Ac-DEVD-pNA (caspase-3 substrate) and Ac-LEHD-pNA (caspase-9 substrate) at 37°C for 2 h. Optical densities were determined at 405 nm using a colorimetric microplate reader. The relative caspase activity was recorded as the ratio to that of the control group.

### RNA Isolation and Real-Time PCR

Total RNA from cells was extracted by Trizol reagent (Invitrogen, 15596018, United States) following the manufacturer’s instructions. Purity of RNA was determined by measuring A260nm/A280nm absorption ratio with ultraviolet spectrophotometer. The cDNA synthesis was generated with Maxima H Minus First Strand cDNA Synthesis Kit with DNase I (Thermo Scientific, #K1682, United States) for reverse transcription according to the manufacturer’s instructions. qRT-PCR was performed using TB GreenTM Premix Ex TaqTM II (Takara, Cat. #RR820A, Japan) on an ABI-7500 Real-time PCR System (Applied Biosystems, United States). GAPDH gene expression was used as internal control for the normalization. A two-step PCR program was performed for gene amplification. The PCR conditions were set to pre-denaturation at 95°C for 30s, followed by 40 cycles at 95°C for 10s and 60°C for 34 s. The 2^−△△*C*t^ method was used to determine the relative gene expression. All primers used in this study are as follows:

**Table 2:** 

Genes	Primers	Sequences
GAPDH	Forward	AGCCTCAAGATCATCAGCAATGCC
GAPDH	Reverse	TGTGGTCATGAGTCCTTCCACGAT
PGC-1α	Forward	CAGAGAGTATGAGAAGCGAGAG
PGC-1α	Reverse	AGCATCACAGGTATAACGGTAG
TFAM	Forward	TTCCAAGAAGCTAAGGGTGATT
TFAM	Reverse	AGAAGATCCTTTCGTCCAACTT
NRF-1	Forward	GCTACTTACACCGAGCATAGTA
NRF-1	Reverse	CTCAAATACATGAGGCCGTTTC
ATP5G1	Forward	GACACAGCAGCCAAGTTTATTG
ATP5G1	Reverse	CCAAGAATGGCATAGGAGAAGA
Cox5a	Forward	GGGTAACATACTTCAACAAGCC
Cox5a	Reverse	AGTTGGTCTAAGTTCCTGGATG
MFN1	Forward	ACAAGGTGAATGAGCGGCTTTCC
MFN1	Reverse	TCTTTCCATGTGCTGTCTGCGTAC
MFN2	Forward	GTGCTTCTCCCTCAACTATGAC
MFN2	Reverse	ATCCGAGAGAGAAATGGAACTC
Drp1	Forward	GAGATGGTGTTCAAGAACCAAC
Drp1	Reverse	CAATAACCTCACAATCTCGCTG
FIS1	Forward	AGTACGCCTGGTGCCTGGTG
FIS1	Reverse	GCTGTTCCTCCTTGCTCCCTTTG
MTFP1	Forward	CCATTGACAAAGGCAAGAAGG
MTFP1	Reverse	CTAGAGCCTGCCATACAAAGG
METTL3	Forward	CTTCAGCAGTTCCTGAATTAGC
METTL3	Reverse	ATGTTAAGGCCAGATCAGAGAG
METTL14	Forward	ACCAAAATCGCCTCCTCCCAAATC
METTL14	Reverse	AGCCACCTCTTTCTCCTCGGAAG
WTAP	Forward	CTGACAAACGGACCAAGTAATG
WTAP	Reverse	AAAGTCATCTTCGGTTGTGTTG
FTO	Forward	GTTCACAACCTCGGTTTAGTTC
FTO	Reverse	CATCATCATTGTCCACATCGTC
ALKBH5	Forward	GCAAGGTGAAGAGCGGCATCC
ALKBH5	Reverse	GTCCACCGTGTGCTCGTTGTAC
YTHDC1	Forward	ATCATCTTCCGTTCGTGCTGTCC
YTHDC1	Reverse	ATACACCCTTCGCTTTGGCAAGAG
YTHDF1	Forward	ATGACAATGACTTTGAGCCCTA
YTHDF1	Reverse	AGGGAGTAAGGAAATCCAATGG
YTHDF2	Forward	ACTTCTCAGCATGGGGAAATAA
YTHDF2	Reverse	TATTCATGCCAGGAGCCTTATT
YTHDF3	Forward	GCTCCACCAACCCAACCAGTTC
YTHDF3	Reverse	CTGAGGTCCTTGTTGCTGCTGTG
MCU	Forward	ATTTGGGAGCTGTTTATTGCAG
MCU	Reverse	GCCTCACAGATATCACAGGTAA

### Cell Apoptosis Analysis

Apoptosis of primary HAECs was detected by flow cytometry according to the manufacturer’s instructions of the FITC Annexin-V apoptosis detection kit (BD pharmingen, United States, cat. No 556547). After various treatments, cells were washed with PBS and digested with 0.25% trypsin without EDTA. Next, cells were centrifuged at 500 *g* for 5 min, washed twice with cold PBS, and resuspended in 100 μl binding buffer. Thereafter, 5 μl of PI and 5 μl of Annexin V-FITC were added and incubated for 15 min in the dark. Finally, 400 μl of binding buffer was added and shaken on an oscillator for a bit. Apoptosis was analyzed using a BD FACSAria™ II cell sorter (BD Biosciences, San Jose, California, United States) at 488 nm.

### RNA m6A Dot Blots

A dot blot assay used to measure the level of mRNA m6A methylation was performed as follows. Total RNA was isolated. The isolated RNA was denatured at 95°C for 3 min, followed by immediate chilling on ice. The denatured RNA (100 ng) was spotted on a Nitrocellulose membrane (GE Healthcare, RPN203B, United States). Then, the membranes were ultraviolet (UV) crosslinked with a StratageneStratalinker 2,400 UV Crosslinker and were blocked in blocking buffer (5% non-fat milk in PBST) for 1 h. N6-Methyladenosine (m6A) recombinant rabbit monoclonal antibody (Invitrogen, MA5-33030, United States) was incubated with the membrane overnight at 4°C with a dilution of 1:1,000. After washing three times with 1 × PBST, a corresponding horseradish peroxidase conjugated secondary antibody (Cell Signaling Technology, 7074S, United States) was diluted 1:5,000 and incubated with the membranes for 1 h at room temperature. After extensive washing, the membrane was visualized by Super-enhanced chemiluminescence detection reagents (Applygen Technologies, P1030, China). To determine whether each dot possess an equal amount of RNA, the RNAs with same amount were spotted on the membrane and stained with 0.02% methylene blue in 0.3 M sodium acetate (pH 5.2) as a control.

### M6a MeRIP-qRT-PCR

MeRIP assays were performed with Magna MeRIP m6A Kit (Millipore, 17-10499, United States) to detect the m6A modification on specific genes according to the manufacturer’s instructions. In brief, 300 μg total RNAs were chemically fragmented into about 300 nucleotides fragments by incubation at 94°C for 3 min in fragmentation buffer (10 mmol/L Tris-HCl, 10 mmol/L ZnCl_2_, and pH 7.0). Then, the RNA samples were immunoprecipitation with anti-m6A antibody (Invitrogen, MA5-33030, United States) or anti-rabbit IgG (Sigma-Aldrich, R2655, United States). The mixed solution was further incubated with pre-washed dynabeads Protein A/G for 2 h at 4°C. The methylated RNAs were eluted with N6-methyladenosine 5′-monophosphate sodium salt (6.7 mM) at 4°C for 1 h and precipitated with 5 mg of glycogen, one-tenth volumes of 3 M sodium acetate in a 2.5 volume of 100% ethanol at −80°C overnight. Further enrichment of m6A was quantified by qRT-PCR and normalized to the input (%Input = 1/10 × 2^*C*t [IP]^−^*C*t [input]^).

### RNA Immunoprecipitation

RNA immunoprecipitation assay was carried out by using Magna RIP RNA-binding Protein immunoprecipitation kit (Millipore Sigma, 17-700, United States) according to the manufacturer’s instructions. Indicated cells were transfected with the same amount of HA-tagged plasmids (HA-YTHDF1, HA-YTHDF2, HA-YTHDF3, HA-△YTH, and HA-YTH) or HA plasmids as a control. All cells were subjected to the same treatment. Cell extracts were prepared with RIP buffer (100 mM KCl, 5 mM MgCl_2_, 1 mM DTT, 10 mM HEPES, and 0.5% NP-40, pH 7.0) and protease inhibitor (Beyotime, China, P1005) and then incubated with 5 μg of normal antibodies against mouse IgG (Millipore Sigma, 12-371, United States), YTHDF1-3, or HA (Abcam, ab18181, United States) overnight at 4°C. RNA enriched by HA RIP of cells expressing untagged protein was served as negative control. Associated RNA-protein complexes were brought down by protein A/G beads. RT-PCR analysis was performed to determine RNA transcripts associated with indicated protein, and the data were normalized to input.

### Statistical Analyses

Results were presented as mean ± standard deviation (SD) and statistical analyses were performed with GraphPad Prism 6 software. Statistical significance was calculated by two-tailed Student’s *t*-test. All experiments were repeated at least three times and a *p* value less than 0.05 was considered to statistically significant.

## Results

### Vitamin D3 Protected HAECs From HCMV-Induced Apoptosis by Inhibiting ER and Mitochondrial Apoptosis Pathway

**Figure 1:**
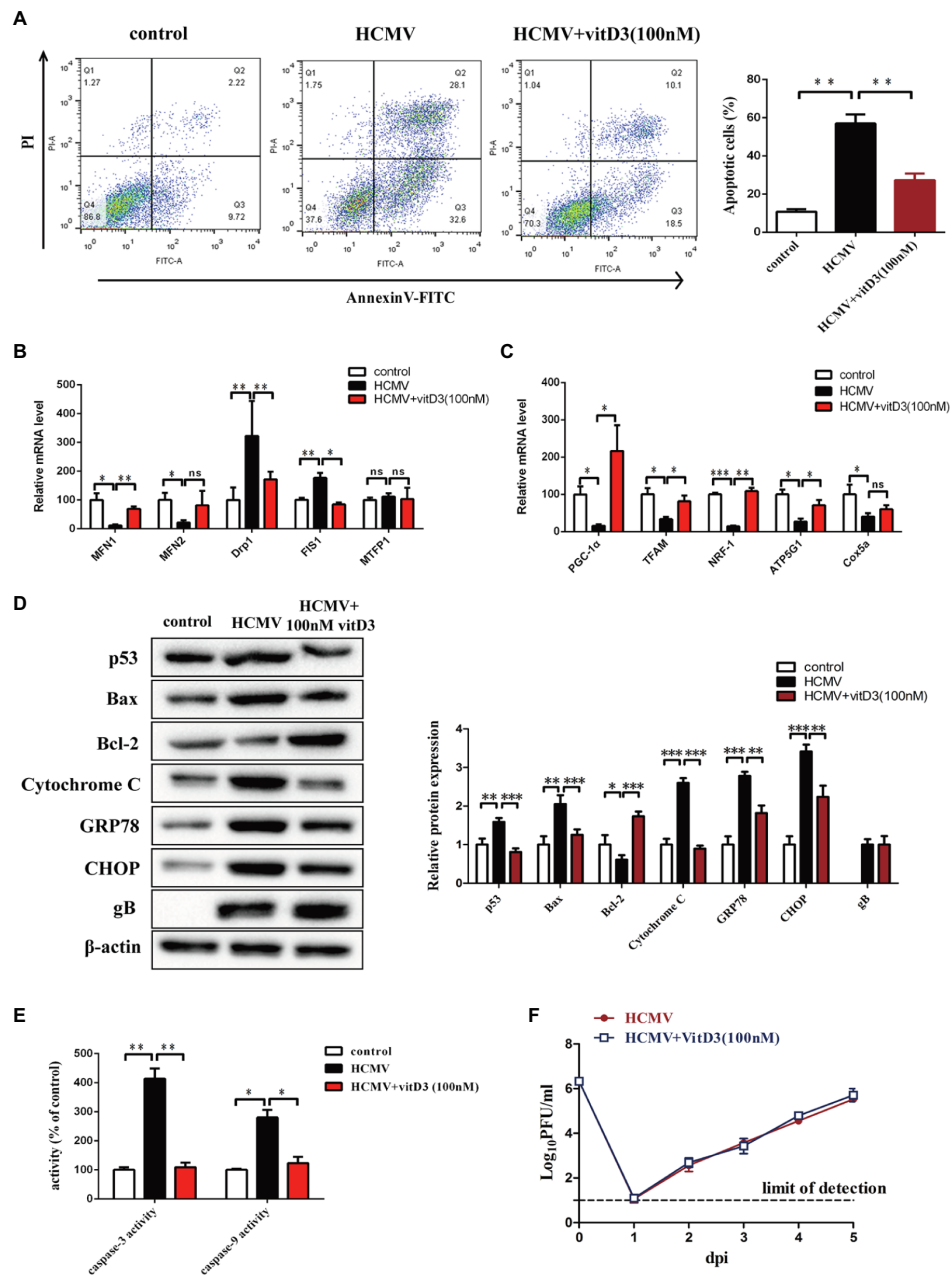
The role of vitD3 on human cytomegalovirus (HCMV)-induced apoptosis of human aortic endothelial cells (HAECs). HAECs were mock-infected (control) or infected with VHL/E strain of HCMV [multiplicity of infection (MOI) = 5] for 4 days. In the vitD3-treated group, HAECs were continuously added with 100 nM vitD3 from the second to the fourth day of HCMV infection. After 4 days, HAECs were harvested for the following experiments. **(A)** Detection of cellular apoptosis in different groups by flow cytometric assay with Annexin-V/PI double staining and quantitative analysis. Left, representative images of flow cytometry; right, statistical charts. Apoptotic cells are calculated as the percentage of Annexin-V single-positive plus Annexin-V/PI double-positive cells. **(B,C)** qPCR analysis of mitochondrial gene expression in the corresponding groups. **(D)** Representative Western blot immunolabeling of proteins representing apoptosis, using β-actin as the loading control. Left, representative graphs of three independent experiments; right, statistical charts with densitometric analysis. gB, a HCMV gene used to quantify HCMV infection. **(E)** The caspase-3 and caspase-9 activities were measured by Caspase-3/9 fluorescent Assay kits. **(F)** Single-cycle viral replication kinetic curves from HAECs infected at an MOI of 5 were plotted by determining the titer in plaque forming unit (PFU) from supernatants collected at the indicated times postinfected. All data are from experiments performed in triplicate and presented as the mean ± standard deviation (SD). Student’s *t-*test, ^*^p < 0.05, ^**^p < 0.01, and ^***^p < 0.001. ns: no significant difference.

To elucidate the protective effect of vitamin D3 on vascular endothelial cells, we constructed a HAECs apoptosis model by infection with HCMV VHL/E strain and detected apoptosis of HAECs by FCM with Annexin V-FITC/PI. As shown in Figure 1A, HCMV infection could obviously increase the ratio of apoptotic cells (60.7%) compared to control (11.94%). When HAECs were co-treated with 100 nM vitamin D3 for 4 days, the ratio of apoptosis induced by HCMV infection was evident decreased (28.6%).

As HCMV proteins can traffic into mitochondria through mitochondria-associated membranes (Williamson and Colberg-Poley, 2009), quantitative PCR assays were performed to evaluate mitochondrial function-related genes. The results showed that the expression of genes regulating mitochondrial fusion (MFN1 and MFN2) was decreased after HCMV infection, while the expression of genes regulating mitochondrial division (Drp1 and FIS1) was upregulated. In contrast, vitamin D3-treated cells exhibited significantly enhanced mitochondrial homeostasis and improved disturbance in mitochondrial division and fusion (Figure 1B). In addition, the genes relate to mitochondrial biogenesis (PGC-1α, TFAM, and NRF1) and oxidative phosphorylation (ATP5G1 and Cox5a) were also decreased after HCMV infection. Vitamin D3 treatment enhanced overall mitochondrial biosynthesis and function of electron transport chain (Figure 1C).

To detect the effect of the mitochondrial apoptotic pathway or ER apoptotic pathway in vitamin D3-mediated protection on HAECs apoptosis induced by HCMV, expression of mitochondrial apoptotic pathway-related protein (Bcl-2, Bax, and cytochrome *c*) and ER apoptotic pathway-related protein (GRP78 and CHOP) were detected by Western blot. As shown in Figure 1D, compared to control group, the Bcl-2 protein expression was obviously decreased, and the expression of p53, Bax, Cytochrome *c*, GRP78, and CHOP was obviously increased in HCMV-infected cells. After addition of 100 nM vitamin D3, Bcl-2 expression was obviously increased, while the expression of mitochondrial apoptosis-related protein (Bax and Cytochrome *c*) or ER apoptosis-related protein (GRP78 and CHOP) was obviously decreased.

The caspase-3 and caspase-9 activities were detected by a fluorescent analysis kit. As shown in Figure 1E, after adding vitamin D3, the caspase-3 and caspase-9 activities were obviously suppressed in HCMV-induced HAECs, indicating apoptosis of HAECs was suppressed by vitamin D3.

To further explore whether the protective effect of vitamin D3 on vascular endothelial cells was related to its effect on HCMV replication, single-cycle viral replication kinetic in HAECs of HCMV single-treatment and vitamin D3 co-treatment groups was plotted. The results showed that the viral replication kinetics were similar in the two groups, and the HCMV replicate indistinguishably in HAECs between HCMV infection alone or with vitamin D3 co-treatment (Figure 1F).

### Vitamin D3 Negatively Regulates the Elevated m6A Modification Levels Induced by HCMV Infection and Inhibits HCMV-Induced Apoptosis by Suppressing METTL3 Expression in HAECs

**Figure 2:**
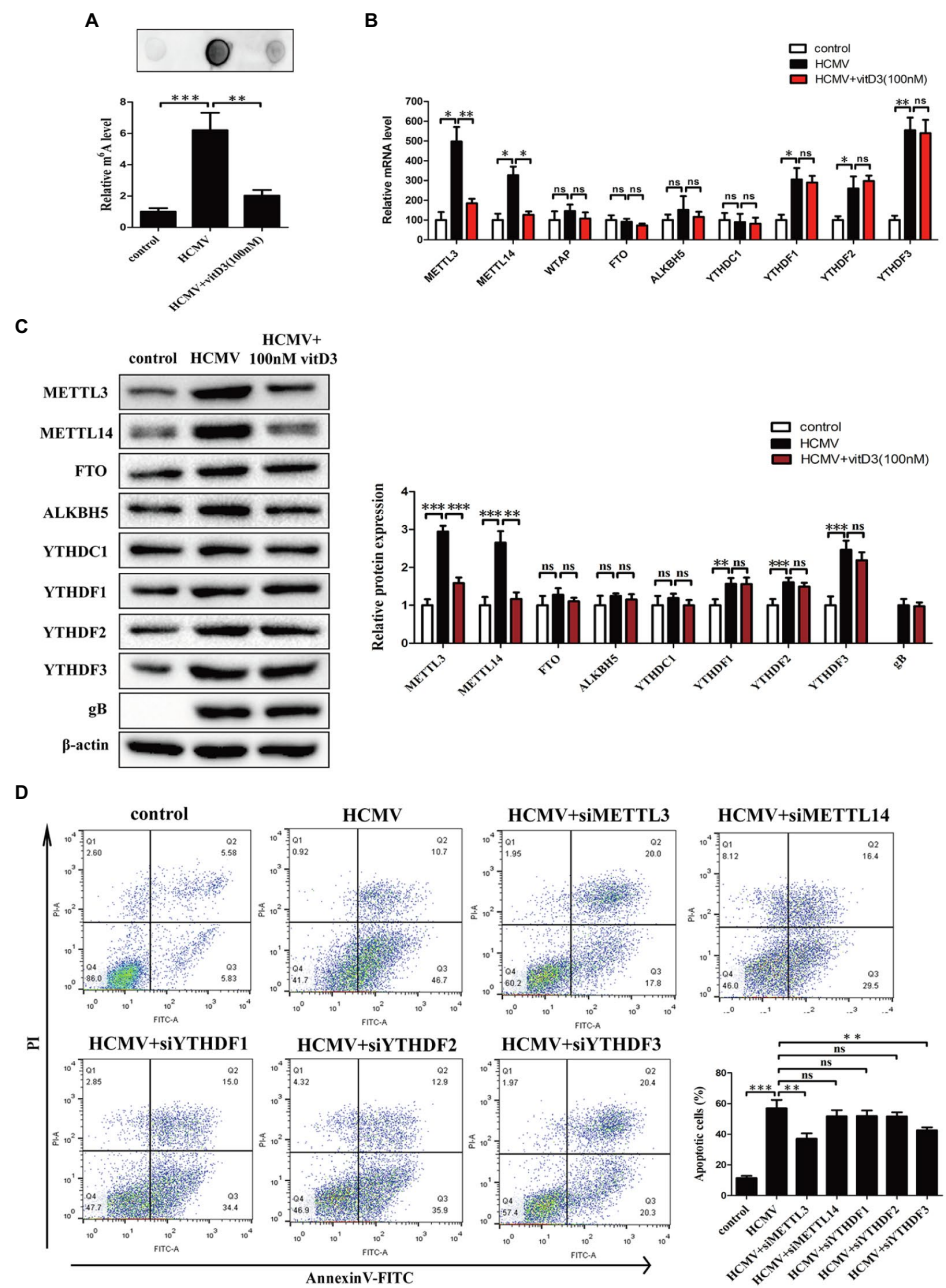
Vitamin D3 negatively regulates the elevated m6A methylation levels induced by HCMV infection and inhibits HCMV-induced apoptosis by suppressing METTL3 expression in HAECs. HAECs were uninfected (control) or infected with VHL/E strain of HCMV [multiplicity of infection (MOI) = 5] for 4 days. In the vitD3-treated group, HAECs were continuously added with 100 nM vitD3 from the second to the fourth day of HCMV infection. After 4 days, HAECs were harvested for the following experiments. **(A)** RNA dot blot analysis of total m6A levels in the corresponding groups. Top, representative images of RNA dot blot; bottom, statistical charts for quantification of RNA dots. **(B)** qPCR analysis of mRNA levels of m6A modification-associated genes. **(C)** Western blot analysis of protein levels of m6A modification-associated genes. Left, representative graphs of three independent experiments; right, statistical charts with densitometric analysis. gB, a HCMV gene used to quantify HCMV infection. **(D)** Apoptosis was detected by Annexin-V/PI-labeled flow cytometric assay in different treatment groups and quantitative analysis. HAECs were transfected with siRNA of the indicated genes for 12 h and then exposed to HCMV. Left, representative images of flow cytometry; right, statistical charts. Apoptotic cells are calculated as the percentage of Annexin-V single-positive plus Annexin-V/PI double-positive cells. All data are expressed as the mean±SD of triplicate experiments. Columns, mean; error bars, SD. Student’s *t-*test, ^*^p < 0.05, ^**^p < 0.01, and ^***^p < 0.001. ns: no significant difference.

Previous study demonstrated that HCMV infection could induce aberrantly elevated m6A modification (Rubio et al., 2018). To determine whether m6A modification was involved in the vitamin D3-mediated protection on HAECs apoptosis induced by HCMV, the levels of m6A modification were measured using m6A mRNA dot blot. Consistent with the results in previous study, the levels of m6A modification were significantly increased in HAECs following HCMV infection. However, the increase in the levels of HCMV-induced m6A modification was inhibited by the addition of vitamin D3 (Figure 2A).

Given that the m^6^A modification is dynamically regulated by methyltransferases-“writers” (METTL3, METTL14, and WTAP), demethylases-“erasers” (ALKBH5 and FTO), and m^6^A binding proteins-“readers” (YTHDF1-3 and YTHDC1), we examined the expression of the key m6A modification-related genes at the mRNA level and protein level by quantitative PCR and Western blot. It was observed that the mRNA levels of METTL3, METTL14, YTHDF1, YTHDF2, and YTHDF3 were significantly increased in HAECs following HCMV infection. However, when the cells were co-treated with vitamin D3, the HCMV-induced elevated mRNA levels of METTL3 and METTL14 were inhibited, but the HCMV-induced elevated mRNA levels of YTHDF1, YTHDF2, and YTHDF3 were not inhibited (Figure 2B). Similar results were observed in protein expression by Western blot (Figure 2C). These results indicate that vitamin D3 negatively regulates HCMV-induced elevated m6A modification by inhibiting the expression of methyltransferases (METTL3 and METTL14).

Since vitamin D3 both inhibits m6A modification and protects arterial endothelial cells from HCMV-induced apoptosis, we hypothesized that the abnormal m6A modification may be involved in HCMV-induced apoptosis. We then assessed the effects of METTL3, METTL14, and YTHDF1-3 silencing on the HCMV-induced apoptosis in HAECs by flow cytometry with Annexin V-FITC/PI double staining. As seen in Figure 2D, the proportion of apoptotic cells during HCMV infection was significantly reduced after METTL3 or YTHDF3 knockdown. In contrast, the amount of HCMV-induced apoptosis was unaffected in the presence of METTL14, YTHDF1, or YTHDF2 silencing (Figure 2D; Supplementary Figures 1A,B). These data suggest that vitamin D3 protects arterial endothelial cells from HCMV-induced apoptosis in part by inhibiting METTL3 expression.

### Vitamin D3 Inhibits HCMV-Induced Apoptosis and Inhibits HCMV-Induce Increase in m6A Methylation *via* the VDR/AMPK Pathway

**Figure 3:**
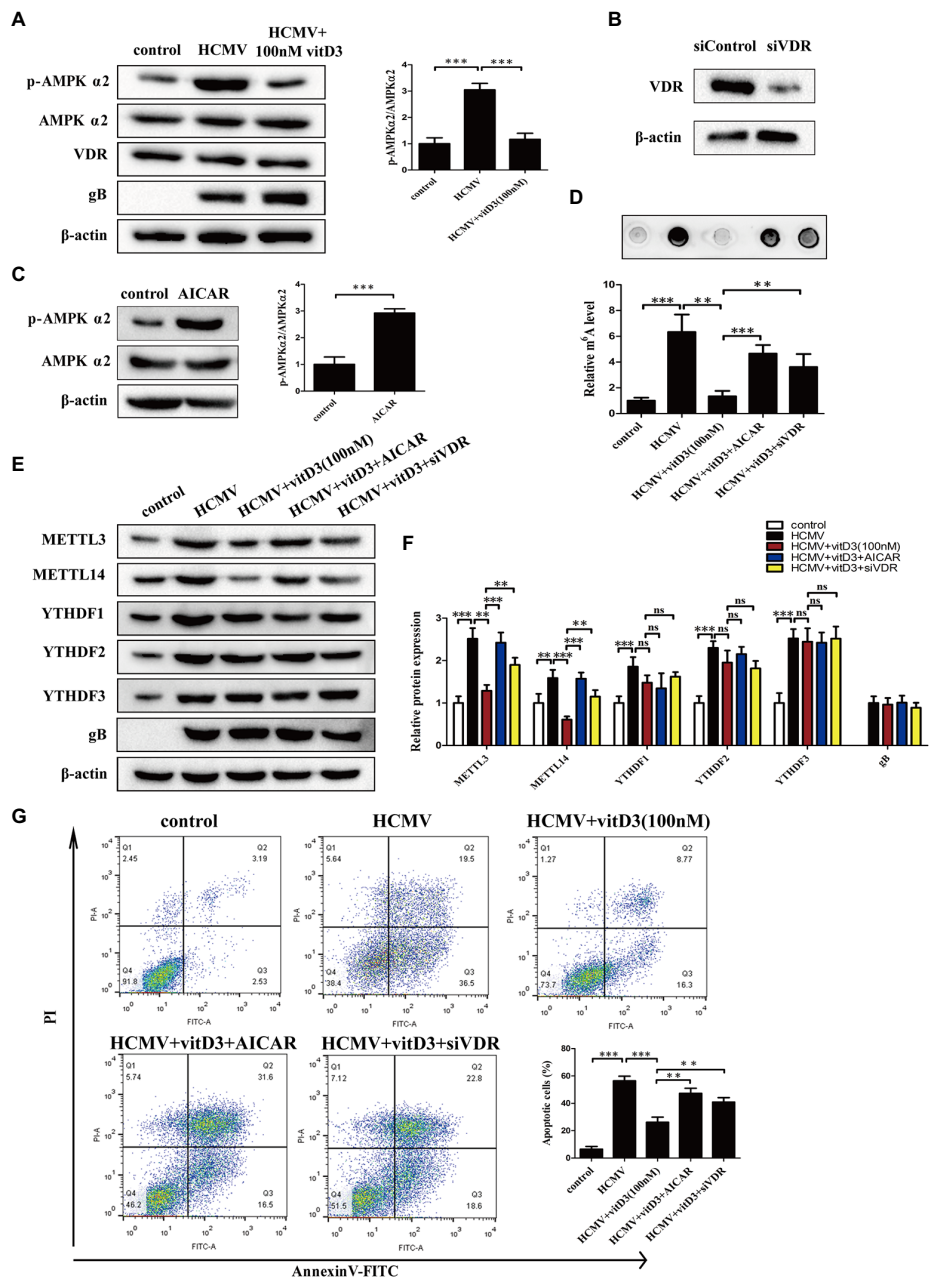
Vitamin D3 inhibits HCMV-induced apoptosis and increased m6A methylation through the VDR/AMPK pathway. **(A)** The expression levels of p-AMPKα2, total AMPKα2, and VDR were detected by Western blotting in HAECs. Left, representative image of three independent experiments; right, statistical histogram representing the ratio of p-AMPKα2 to total AMPKα2. gB, a HCMV gene used to quantify HCMV infection. **(B)** Western blot analysis for knockdown efficiency of VDR in HAECs with siRNA (siVDR). **(C)** Western blot analysis to verify the effect of AMPK activator (AICAR) to increase p-AMPK levels. Left, representative image of triplicate experiments; right, statistical histogram representing the ratio of p-AMPKα2 to total AMPKα2. **(D)** RNA dot blot analysis verifies that vitD3 inhibits the HCMV-induced increase in total m6A levels through the VDR/AMPK pathway. HAECs were exposed to HCMV and simultaneously treated with vitD3 (100 nM) and AICAR (1 mM) for 4 days, or HAECs were transfected with siVDR for 12 h and then exposed to HCMV and vitD3. Top, representative images of RNA dot blot; bottom, statistical charts for quantification of RNA dots. **(E)** Representative Western blotting graphs verify that VitD3-induced alterations in m6A modification-associated proteins are *via* the VDR/AMPK pathway. **(F)** Histogram representing a densitometric analysis performed to quantify the relative intensity of the indicated bands detected by Western blotting. **(G)** Apoptosis rate detected by Annexin-V/PI-labeled flow cytometric assay in different treatment groups shows that vitD3 inhibits HCMV-induced apoptosis through VDR/AMPK pathway. Left, representative images of flow cytometry; right, statistical charts. All data are presented as the mean ± SD of triplicate experiments. Student’s *t-*test, ^**^p < 0.01 and ^***^p < 0.001. ns: no significant difference.

As we all know, vitamin D receptor signaling plays a key role in mediating the effect of vitamin D3. Vitamin D3 and its metabolites regulate the transcription of target genes and exert biological effects through the VDR. Furthermore, AMPK phosphorylation is regulated by Vitamin D3 *via* VDR and plays a protective role in a variety of pathological processes (Choi et al., 2013; Chang and Kim, 2019). Thus, to better understand how vitamin D3 inhibits HCMV-induced apoptosis and inhibits HCMV-induced increase in m6A modification, these two upstream pathway molecules were analyzed. First, we examined whether AMPK phosphorylation and VDR expression were altered under HCMV infection with or without vitamin D3 treatment. As shown in Figure 3A, AMPKα2 phosphorylation was obviously increased in the HCMV-infected group compared to the control group, yet significantly decreased after vitamin D3 co-treatment. In contrast, total AMPK protein levels and VDR expression did not change under HCMV infection with or without vitamin D3 treatment. These findings indicate that vitamin D3 treatment attenuated the increase in AMPK phosphorylation.

To directly address the role of AMPK or VDR on the effect of vitamin D3, we treated HAECs with AMPK activator (AICAR) or siVDR. The knockdown efficiency of VDR was shown in Figure 3B. In addition, activation of AMPK by AICAR upregulated the p-AMPKα2 level (Figure 3C). We then assessed the role of VDR and AMPK in vitamin D3-mediated inhibition of m6A modification by m6A mRNA dot blot and Western blot assays. Consistent with the previous results, the m6A mRNA dot blot assays showed that vitamin D3 treatment attenuated the increase in HCMV-induced m6A modification. However, when VDR was absent or the activator of AMPK (AICAR) was added, the level of HCMV-induced m6A modification was again increased (Figure 3D). Western blot assays showed the similar results, vitamin D3 treatment attenuated the HCMV-induced increase in the expression of m6A modification-related proteins (METTL3 or METTL14). However, when VDR was deleted or AMPK activator (AICAR) was added, the effect of vitamin D3 was diminished and METTL3 or METTL14 expression was elevated again. In contrast, YTHDF1-3 expression was increased under HCMV infection but was not affected by vitamin D3, VDR, and AMPK activity (Figures 3E,F).

To determine the role of VDR and AMPK in vitamin D3-mediated inhibition of apoptosis induced by HCMV infection, flow cytometry with Annexin V-FITC/PI double staining was performed. As shown in Figure 3G, vitamin D3 treatment protected the HAECs from HCMV-induced apoptosis, and AICAR co-treatment or VDR deletion rose the percentage of apoptosis again. Taken together, these data suggest that vitamin D3 inhibits HCMV-induced apoptosis and HCMV-induce increase in m6A methylation *via* the VDR/AMPK pathway.

### Vitamin D3 Reduces the HCMV-Induced Increase in MCU Expression *via* the VDR/AMPK Pathway and MCU Contributes To HCMV-Induced Apoptosis in HAECs

**Figure 4:**
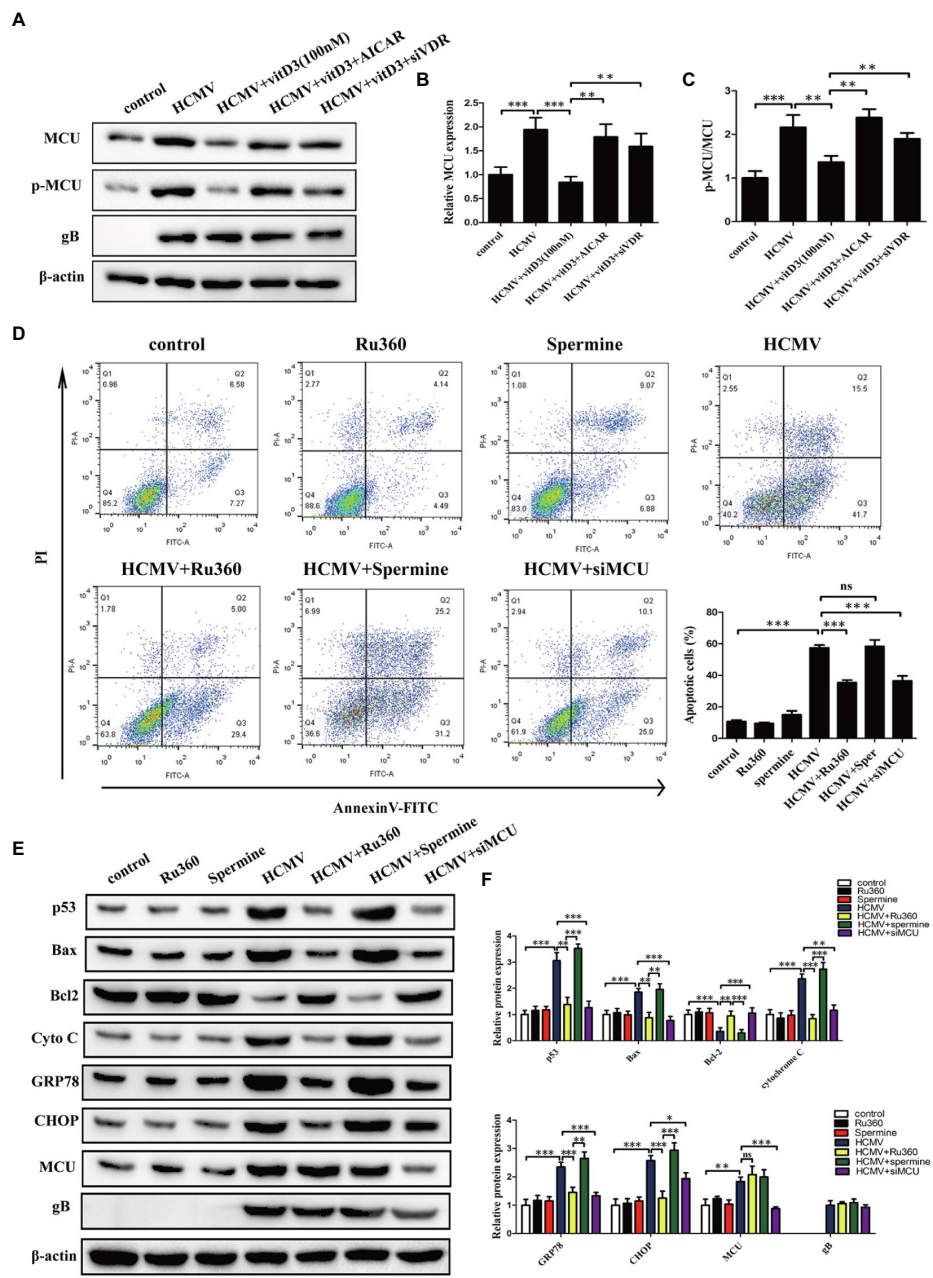
Vitamin D3 reduces MCU expression *via* the VDR/AMPK pathway and MCU contributes to HCMV-induced apoptosis. **(A)** Representative Western blotting graphs verify that vitD3 reduces HCMV-induced increase in MCU (phosphorylated and total MCU) expression *via* the VDR/AMPK pathway. **(B)** Statistical histogram representing a densitometric analysis performed to quantify the relative intensity of the total MCU expressive bands detected by Western blotting in **(A)**. **(C)** Statistical histogram representing a densitometric analysis performed to quantify the ratio of p-MCU to total MCU in **(A)**. **(D)** Representative Annexin-V/PI double-labeled flow cytometric assay shows that MCU inhibition attenuates the apoptosis induced by HCMV. Left, representative images of flow cytometry; right, statistical charts. **(E)** Representative Western blotting graphs verify that MCU inhibition attenuates the expression of apoptosis-associated proteins. HAECs were exposed to HCMV or not with or without Ru360 (5 μM) or spermine (10 μM). gB, a HCMV gene used to quantify HCMV infection. **(F)** Statistical histogram representing a densitometric analysis performed to quantify the relative intensity of the indicated bands detected by Western blotting in **(E)**. All data are expressed as the mean ± SD of triplicate experiments. Student’s *t-*test, ^*^p < 0.05, ^**^p < 0.01, and ^***^p < 0.001. ns: no significant difference.

Given the known role of the mitochondrial protein MCU in regulating apoptosis by modulating mitochondrial function and ER stress (Guan et al., 2019; Li et al., 2020), we hypothesized that MCU may contribute to HCMV-induced apoptosis. To test this hypothesis, we first examined the expression levels of MCU under different treatment conditions. It was observed that both total MCU protein and phosphorylated MCU protein were significantly increased after HCMV infection, and this increase was reversed after the addition of vitamin D3 and restored after exposure to AICAR or siVDR (Figures 4A–C). This finding indicated that vitamin D3 could reduce the HCMV-induced increase in total and phosphorylated MCU expression *via* the VDR/AMPK pathway.

To further determine whether MCU contributes to HCMV-induced apoptosis in HAECs, cellular apoptosis was measured using flow cytometry with Annexin V-FITC/PT double staining. We introduced Ru360 as MCU inhibitor or spermine as MCU activator, which has no effect on apoptosis of HAECs in normal state. HCMV infection induced a high percentage of apoptosis, which could be significantly reduced by MCU inhibition (Ru360 or siMCU) and further increased with spermine (Figure 4D; Supplementary Figure 1C). Additionally, apoptosis was estimated by apoptosis-related proteins including p53, Bax, Bcl-2, cytochrome *c*, GRP78, and CHOP by Western blot. In coincidence with flow cytometry results, HCMV infection induced the increment of p53, Bax, cytochrome *c*, GRP78, and CHOP, and the decrement of Bcl-2, and Ru360 or siMCU dampened these apoptotic changes while spermine enhanced apoptosis (Figures 4E,F).

The caspase-3 and caspase-9 activities were detected by a fluorescent analysis kit. As shown in Supplementary Figure 1D, the caspase-3 and caspase-9 activities were obviously increased under HCMV infection. This change could be reversed by MCU inhibition (Ru360 or siMCU) or be accelerated by MCU activator (spermine). These data confirm that MCU was upregulated during HCMV infection and contributed to HCMV-induced apoptosis.

### METTL3 Upregulates MCU Protein Expression (but Not mRNA) by Catalyzing Its m6A Methylation in Mock-Infected or HCMV-Infected HAECs

**Figure 5:**
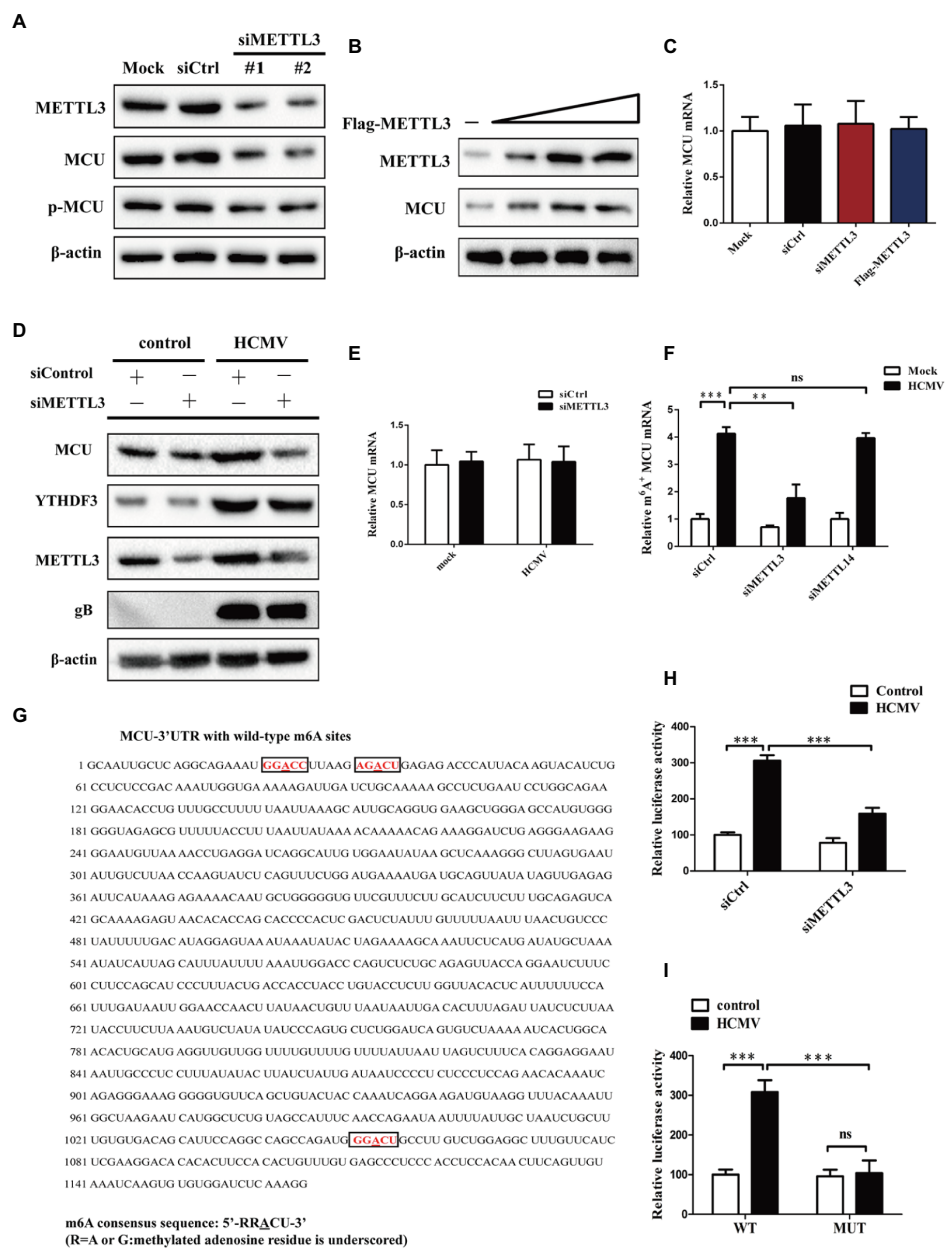
METTL3 upregulates MCU protein expression (but not mRNA) by catalyzing its m6A methylation in mock-infected or HCMV-infected HAECs. **(A)** Western blot analysis of MCU protein levels after knockdown of METTL3 in mock-infected HAECs. **(B)** Western blot analysis of MCU protein levels after overexpression of METTL3 in mock-infected HAECs. HAECs were transfected with a different dose of METTL3 expression plasmids and then subjected to immunoblot. **(C)** qRT-PCR analysis to quantify the relative MCU mRNA level after knockdown or overexpression of METTL3 in mock-infected HAECs. **(D)** Western blot analysis of MCU protein level after METTL3 knockdown in HCMV-infected HAECs. **(E)** qRT-PCR analysis to quantify the relative MCU mRNA level after knockdown of METTL3 in HCMV-infected HAECs. **(F)** m6A immunoprecipitation and qRT-PCR were performed to determine the change of MCU mRNA with m6A methylation. HAECs with or without knockdown of METTL3 or METTL14 were exposed to HCMV. **(G)** Sequence analysis of the MCU 3′-UTR revealed three matches to the 5′-RRACU-3′ (underlined are methylated adenosine residue) m6A consensus sequence. **(H)** Luciferase assays were performed by transfecting HAECs with a reporter plasmid containing luciferase coding sequences followed by the MCU 3′-UTR or empty vector only containing luciferase. The ratio indicating the luciferase activity in cells transfected with MCU 3′-UTR relative to empty vector was determined. **(I)** Luciferase assays were performed in HAECs transfected with WT or mutant (MUT) luciferase-MCU 3′-UTR reporters. The ratio of luciferase activity in cells transfected with MCU-3′UTR relative to empty vector was determined. All data are expressed as the mean ± SD of triplicate experiments. Student’s *t-*test, ^**^p < 0.01 and ^***^p < 0.001. ns: no significant difference.

Since HCMV infection induced both elevated levels of m6A modification and MCU expression, and both m6A modification and MCU contribute to HCMV-induced apoptosis, we wondered whether the altered MCU expression was the consequence of HCMV-mediated m6A methylation modification. First, we investigated whether METTL3 or METTL14 (two m6A methyltransferases upregulated by HCMV infection) regulate MCU expression. It was observed that siRNA-mediated knockdown of METTL3 in HAECs significantly reduced MCU protein levels, while having no effect on p-MCU expression (Figure 5A). Conversely, we observed a dose-dependent increase in MCU protein levels in HAECs in response to METTL3 overexpression (Figure 5B). However, there was no change in MCU mRNA levels in HAECs either by knockdown of METTL3 or overexpression of METTL3 (Figure 5C). In contrast, METTL14 had no regulatory effect on MCU expression, as MCU protein expression was unchanged by either knocking down METLL14 or overexpressing METTL14 (Supplementary Figures 1E,F). In addition, knockdown of METTL3 in HCMV-infected HAECs similarly reduced the increase in protein expression of MCU induced by HCMV infection (mRNA expression was not affected; Figures 5D,E).

To further test the hypothesis that altered MCU expression was the consequence of METTL3-mediated m6A methylation modification, total cellular RNA was immunoprecipitated with an antibody recognizing m6A, and qRT-PCR was performed on the immunoprecipitated RNA to amplify the MCU 3′-UTR. We found that HCMV exposure led to an increase in MCU mRNA m6A methylation levels in HAECs. However, silencing METTL3 but not METTL14 resulted in decreased MCU m6A methylation levels under HCMV infection conditions, suggesting that MCU mRNA may be subject to METTL3-dependent m6A methylation (Figure 5F).

To further demonstrate that MCU mRNA is a direct target of METTL3-dependent m6A methylation, we searched for sequences within the MCU 3′-UTR. Sequence analysis of the MCU 3′-UTR revealed three matches to the 5′-RRACU-3′ (methylated adenosine residue is underlined) m6A consensus sequence (Figure 5G). In addition, a luciferase reporter plasmid that contained the entire MCU 3′-UTR was transiently transfected into HAECs, followed by exposure to HCMV infection and measurement of luciferase activity (Figure 5H). It was observed that HCMV infection significantly increased luciferase activity, whereas this effect was rescued by METTL3 knockdown in HAECs. Next, the adenine residues of the m6A consensus sequence in the MCU 3′-UTR were mutated (5′-GGACU-3′ to 5′-GGTCU-3′ of mRNA; Supplementary Figure 1G), and the WT and mutant reporters were compared by luciferase assay. Mutations of the adenosine residues in three identified m6A-consistent sequences lead to a decrease in luciferase activity in HAECs, suggesting that the mutation prevented m6A methylation and consequently reduced the translation efficiency of the luciferase-MCU-3′-UTR fusion mRNA (Figure 5I). Taken together, these results presented in Figure 5 indicate that HCMV infection-induced METTL3 expression increases MCU mRNA methylation and translation efficiency.

### YTHDF3 Binds to MCU mRNA and Promotes MCU Protein Translation Through a METTL3-Mediated m6A-Dependent Manner

**Figure 6:**
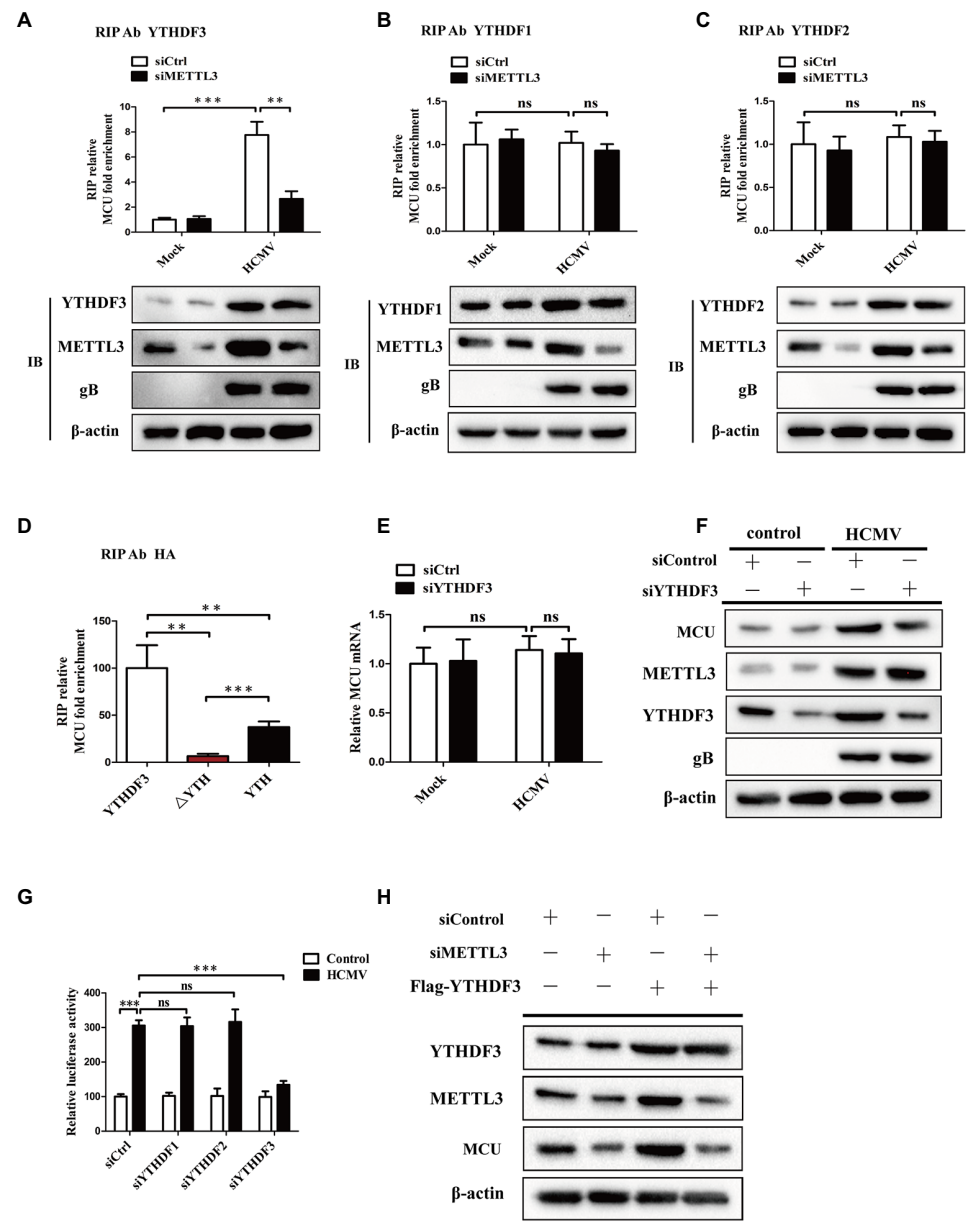
YTHDF3 promotes translation of MCU protein by binding to the MCU mRNA dependently of METTL3-mediated m6A modification. **(A–C)**, RIP analysis of the interaction of YTHDF3 **(A)** or YTHDF1 **(B)** or YTHDF2 **(C)** with MCU mRNA using total cell lysates of HAECs with or without METTL3 knockdown under mock-infected or HCMV-infected condition. Enrichment of MCU mRNA with an antibody against YTHDF3 or YTHDF1 or YTHDF2 was measured by qRT-PCR and normalized to input. Western blotting was performed as indicated. **(D)** MCU RNA enrichment by HA RIP qPCR. Cells were transfected with HA-YTHDF3, HA-△YTH, or HA-YTH, then exposed to HCMV infection. Magnetic beads coated with 5ug of antibody against HA were incubated with indicated cell lysates. **(E)** qRT-PCR analysis of MCU mRNA in HAECs with or without YTHDF3 knockdown under mock-infected or HCMV-infected condition. **(F)** Western blotting of MCU protein expression in HAECs with or without YTHDF3 knockdown under mock-infected or HCMV-infected condition. **(G)** Luciferase assays analysis of changes in translation efficiency of luciferase-MCU 3′-UTR mRNA by knockdown of YTHDF1-3. **(H)** Western blotting analysis to identify whether the impact of YTHDF3 on MCU protein expression was dependent on METTL3. Cells were transfected with Flag-YTHDF3 with or without METTL3 knockdown. All data are expressed as the mean ± SD of triplicate experiments. *p* values were calculated with student’s *t-*test, ^**^p < 0.01 and ^***^p < 0.001. ns: no significant difference.

It was observed that the levels of m^6^A binding proteins-“readers” (YTHDF1-3) were elevated in response to HCMV infection in our previous results. Importantly, YTHDF1-3 reportedly regulates mRNA or protein expression and have been shown to bind to m6A-modified RNA (Fu et al., 2014; Wang et al., 2015; Zhang et al., 2019, 2020). To further explain the correlation between m6A methylation and MCU abundance, we investigated whether YTHDF1-3 was involved in the METTL3-mediated increase in translation of MCU mRNA. We first tested whether YTHDF1-3 interacted with MCU mRNA. RIP-qRT-PCR experiments showed that YTHDF1-3 all interacted with MCU mRNA. HCMV infection not only upregulated YTHDF3 expression but also promoted its binding to MCU mRNA, and this interaction was significantly decreased after METTL3 knockdown (Figure 6A). In contrast, silencing METTL3 did not affect the association of YTHDF1 or YTHDF2 with MCU mRNA (Figures 6B,C). Moreover, to identify the MCU RNA-binding domain of YTHDF3, we stably expressed YTHDF3 deletion mutants in HAECs followed by exposure to HCMV infection. We found that only full-length YTHDF3 could bind to MCU mRNA. Deletion of the YTH domain (△YTH) of YTHDF3 abrogated the binding to MCU mRNA. MCU mRNA was enriched by the YTH domain by about 5-fold more than the △YTH mutant, but YTH alone did not regain the full ability of binding target RNAs (Figure 6D).

We then investigated whether YTHDF3 could regulate MCU expression by regulating the translation of MCU mRNA. As shown in Figures 6E,F, YTHDF3 knockdown attenuated the MCU protein expression induced by HCMV infection but did not affect the MCU mRNA levels. The luciferase assays also showed decreased MCU 3′-UTR luciferase activity in HAECs after YTHDF3 knockdown, but not in YTHDF1 or YTHDF2 (Figure 6G). In addition, elevated MCU protein expression was observed in YTHDF3 overexpressed HAECs and this increase was significantly reduced by METTL3 knockdown (Figure 6H). These data suggest that YTHDF3 plays a vital role in the regulation of MCU expression by promoting MCU protein translation *via* a METTL3-mediated m6A-dependent manner.

### ALKBH5 Not FTO Reverses HCMV-Mediated m6A Modification of MCU mRNA in HAECs

**Figure 7:**
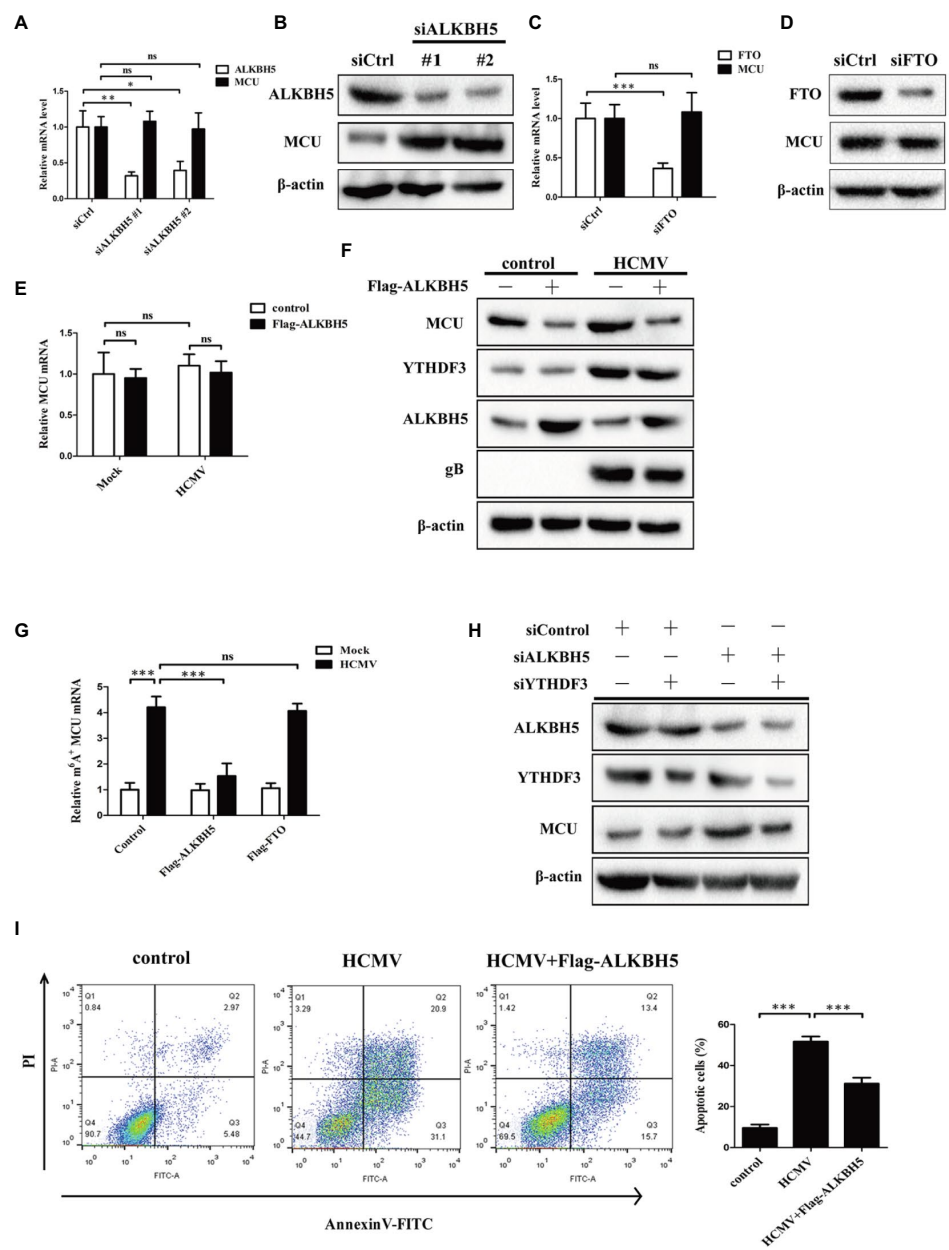
ALKBH5, but not FTO, reverses HCMV-mediated m6A modification of MCU mRNA in HAECs. **(A)** qRT-PCR analysis of MCU mRNA expression in HAECs with or without ALKBH5 knockdown. Samples were normalized to GAPDH mRNA. **(B)** Representative Western blotting of MCU in HAECs with or without ALKBH5 knockdown. **(C)** qRT-PCR analysis of MCU mRNA expression in HAECs with or without FTO knockdown. Samples were normalized to GAPDH mRNA. **(D)** Representative Western blotting of MCU protein expression in HAECs with or without FTO knockdown. **(E)** qRT-PCR analysis of MCU mRNA expression in HAECs with or without ALKBH5 overexpression under mock-infected or HCMV-infected condition. **(F)** Representative Western blotting of MCU protein expression in HAECs with or without ALKBH5 overexpression under mock-infected or HCMV-infected condition. **(G)** m6A immunoprecipitation and qRT-PCR were performed to identify changes in MCU mRNA that underwent m6A methylation modification in HAECs with or without overexpression of ALKBH5 or FTO under mock-infected or HCMV-infected condition. **(H)** Western blotting analysis was performed to determine whether the effect of ALKBH5 on MCU protein expression was dependent on the promotion of MCU translation by YTHDF3. **(I)** Annexin-V/PI double-labeled flow cytometric assay shows that ALKBH5 overexpression attenuates the apoptosis induced by HCMV. Left, representative images of flow cytometry; right, statistical charts. All data are expressed as the mean ± SD of triplicate experiments. Values of *p* were calculated with student’s *t-*test, ^*^p < 0.05, ^**^p < 0.01, and ^***^p < 0.001. ns: no significant difference.

In mammalian cells, m6A methylation modification is a dynamic and reversible process. ALKBH5 and FTO, as two mammalian RNA demethylases, can reverse m6A methylation in cells (Meyer and Jaffrey, 2017). Although our previous results showed that the expression of ALKBH5 and FTO did not change after HCMV infection, we still wondered whether ALKBH5 or FTO was involved in the HCMV-mediated m6A methylation of MCU mRNA. We first measured the change in total mRNA and protein levels of MCU upon ALKBH5 knockdown in HCMV uninfected HAECs. The results showed that knockdown of ALKBH5 significantly increased protein abundance of MCU, but not mRNA (Figures 7A,B). In contrast, FTO knockdown did not change the expression of MCU mRNA and protein, suggesting that MCU may not be a direct target of FTO in HAECs (Figures 7C,D). Next, we tested the change in total mRNA and protein levels of MCU upon ALKBH5 overexpression in HCMV-infected HAECs. It was also observed that ALKBH5 overexpression strongly reduced the increased levels of MCU protein induced by HCMV infection but did not affect the total MCU mRNA (Figures 7E,F). These data suggest that MCU mRNA may be a substrate for ALKBH5 demethylase and this demethylase may not affect the stability but affect the translation efficiency of MCU mRNA.

To further investigate this idea, we performed methylated RNA immunoprecipitation (MeRIP) with m6A antibody of HCMV-infected HAECs in combination with qRT-PCR to determine the levels of MCU m6A methylation following ALKBH5 overexpression. Our results confirmed that m6A methylation on MCU mRNA was strongly increased in response to HCMV infection, whereas ALKBH5 overexpression significantly reduced the increased m6A levels of MCU mRNA induced by HCMV and this reduction was not observed by FTO overexpression (Figure 7G). These data indicate that ALKBH5 mainly affects MCU expression through its demethylation activity. As ALKBH5 can regulate MCU protein levels without effect on MCU mRNA, we wondered whether YTHDF3 was involved in the regulation of MCU protein by ALKBH5. Our experiments showed that the increased levels of MCU protein induced by ALKBH5 knockdown were significantly reduced by simultaneously silencing the YTHDF3 (Figure 7H), suggesting that the regulation of MCU protein by ALKBH5 is dependent on the effect of YTHDF3 on MCU mRNA translation process.

Next, the effects of ALKBH5 on apoptosis in HCMV-infected HAECs were examined. As shown in Figure 7I, the overexpression of ALKBH5 inhibited the apoptosis of HCMV-infected HAECs.

**Figure 8:**
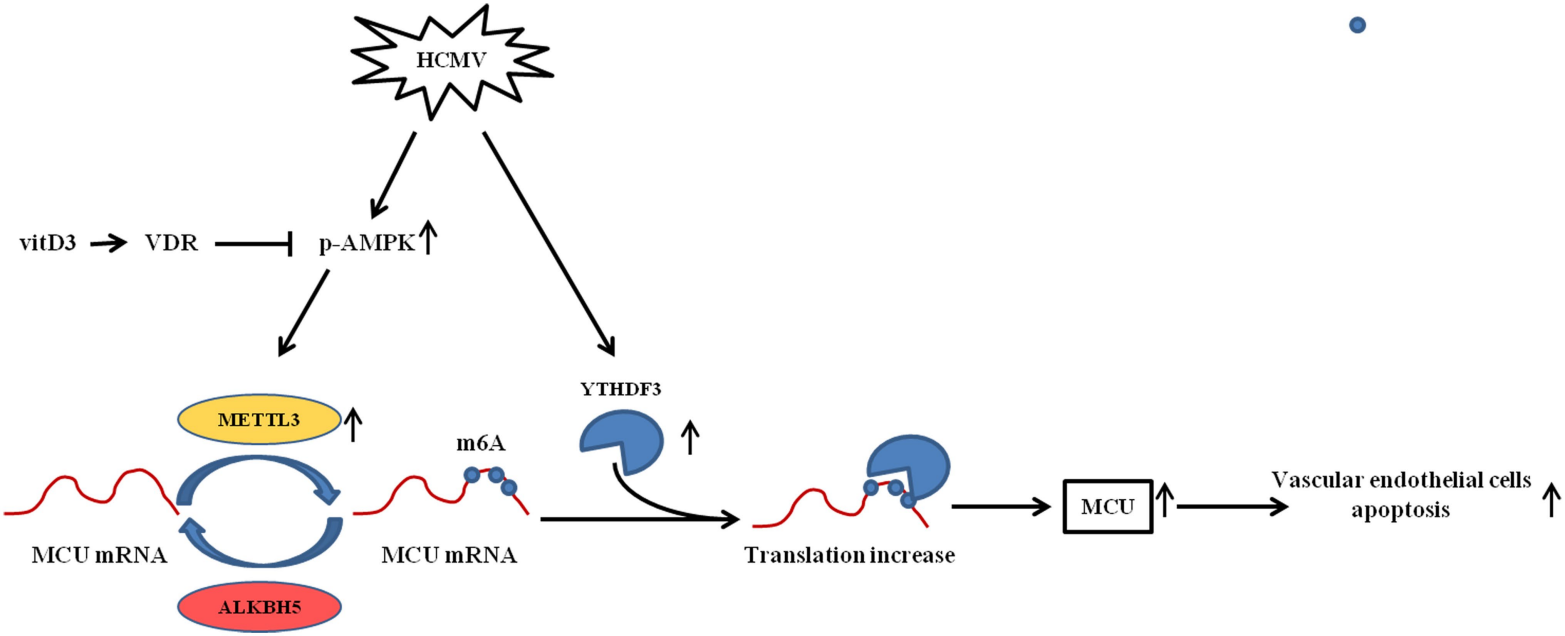
The mechanism diagram of vitamin D3 inhibiting HCMV-induced apoptosis in HAECs. HCMV infection upregulates the expression of METTL3 and YTHDF3. METTL3 methylates MCU at three m6A residues in the 3′-UTR, thereby promoting the binding of RNA-binding protein YTHDF3 to MCU-methylated mRNA and promoting the translation of MCU mRNA. Increased MCU protein expression is responsible for HCMV-induced apoptosis. Vitamin D3 inhibits METTL3 expression *via* the VDR/AMPK pathway, thereby suppressing the MCU m6A modification and MCU-induced apoptosis.

Based on the above results, as shown in Figure 8, it was concluded that HCMV induces apoptosis of vascular endothelial cells by promoting increased translation of MCU *via* METTL3- and YTHDF3-dependent m6A methylation mechanisms. In contrast, vitamin D3 downregulates the METTL3 by inhibiting the activation of AMPK, thereby inhibiting the m6A modification of MCU and cell apoptosis.

## Discussion

Growing evidence indicates that HCMV infection may be the initiating factor of atherosclerosis and that it plays important roles in the occurrence and development of atherosclerosis (Hsich et al., 2001; Jia et al., 2017; Zhu and Liu, 2020). In the process of HCMV-induced atherosclerosis, the dysfunction and even apoptosis of vascular endothelial cells are the distinctive features (Shen et al., 2004; Utama et al., 2006). Since there is no specific drug to clear HCMV infection, there is an urgent need to find effective drugs to treat HCMV infection-related vascular endothelial cell damage. In the present study, we found that vitamin D3 could protect vascular endothelial cells from HCMV-induced apoptosis. Further exploring the mechanisms involved, we found that HCMV infection leads to an overall increase in the level of m6A modification in vascular endothelial cells. Moreover, it was found that HCMV infection could promote vascular endothelial cell apoptosis by increasing MCU protein expression in an m6A-mediated pattern. Further identification revealed that METTL3 is the methylation enzyme of MCU mRNA and YTHDF3 is the “reader” of MCU m6A locus. YTHDF3 can bind to the 3′UTR of MCU that undergoes m6A modification and promote the translation efficiency of MCU protein. Vitamin D3, on the other hand, can inhibit AMPK activation through VDR and reduce METTL3 expression (a downstream target of AMPK), thereby reducing MCU protein expression and ameliorating HCMV-induced apoptosis in vascular endothelia cells.

Vitamin D3 receptor (VDR), a member of the nuclear receptor family of transcription factors, is widely expressed on vascular tissues, such as vascular endothelial cells and vascular smooth muscle cells (Latic and Erben, 2020). This suggests that vitamin D3 plays an important role in the regulation of physiological function of blood vessels. A growing body of evidence suggests that vitamin D3 protects endothelial cells function from radiation, immune damage, and oxidative stress-induced injury (Dehghani et al., 2013; Polidoro et al., 2013; Marampon et al., 2016). In line with the above study, this study also found that vitamin D3 produced a protective effect on vascular endothelial cells against damage caused by HCMV infection. In addition, this anti-apoptotic ability was not due to inhibition of HCMV viral replication. Although it has been reported in the literature that vitamin D3 induces HCMV lytic replication marked by upregulation of HCMV gene expression and production of infectious virus (Wu and Miller, 2015), our experiments did not find the vitamin D3 could affect HCMV replication in vascular endothelial cells. Signaling analysis showed that vitamin D3 could downregulate the elevated level of p-AMPK induced by HCMV infection. Treatment with AICAR (an AMPK activator) or knockdown of the VDR attenuated the anti-apoptotic ability of vitamin D3 in HCMV-infected vascular endothelial cells, suggesting that vitamin D3 exerts its endothelial protective function through the VDR/AMPK axis.

As the most prevalent and abundant RNA modification in eukaryotes, N6-methyladenosine (m6A) modification has also attracted increasing attention in the regulation of cell fate (e.g., apoptosis; Shen et al., 2021; Yuan et al., 2021). Previous study showed that the overall abundance of cellular m6A writers METTL3/14, erasers ALKBH5 and FTO, and reader proteins increased in response to HCMV infection in normal human dermal fibroblasts (NHDFs; Rubio et al., 2018). Our experiments saw consistent results in vascular endothelial cells, with a slight difference that we did not see an increase in the demethylases ALKBH5 and FTO after HCMV infection. To understand the role of m6A modification in HCMV-induced apoptosis in endothelial cells, we knocked down the m6A writers METTL3/14 and the reader protein. We found that knockdown of METTL3 and YTHDF3 alleviated HCMV-induced apoptosis, suggesting that METTL3 and YTHDF3 mediated HCMV-triggered apoptosis in endothelial cells. In addition, vitamin D3 treatment downregulated the elevated expression of METTL3/14 induced by HCMV. However, this downregulation was reversed when AICAR was co-added, indicating that METTL3/14 is a downstream target of the AMPK pathway.

Aberrant expression or phosphorylation modification status of MCU causes disorder of the mitochondrial Ca^2+^ transients, leading to cell death (Guan et al., 2019; Zhao et al., 2019; Li et al., 2020). In our present study, elevated amounts of total and phosphorylated proteins of MCU triggered by HCMV infection were also observed. Intervention with Ru360, an inhibitor of MCU function, or knock down the expression of MCU did attenuate HCMV-induced apoptosis. Further exploration indicated that treatment with vitamin D3 could downregulate the elevated level of MCU induced by HCMV *via* VDR/AMPK axis. Since inhibition of AMPK activation by vitamin D3 can reduce both METTL3/14 and MCU expression, we speculate that m6A modification may be involved in the regulation of MCU expression. Our study demonstrated that the methyltransferase METTL3, but not METTL14, mediated the methylation modification of MCU. Despite the identification of the biological significance of METTL3-mediated m6A modification, the mechanism of how m6A regulates MCU expression remains elusive. RNA metabolism regulation of m6A-modified mRNAs by so-called m6A readers is a fascinating field that has just started to be unveiled recently. The m6A readers, such as the YTH domain-containing proteins (YTHDF1, 2, and 3), selectively bind to m6A-modified RNAs and play a distinctive function. YTHDF2 has been implicated in RNA transport, instability, and degradation (Fu et al., 2014). While YTHDF1 and YTHDF3 can promote translation of m6A-modified mRNA through an interaction with the ribosomal proteins of 40S/60S subunits (Wang et al., 2015; Li et al., 2017). Since all three readers were increased in HCMV-infected endothelial cells, we identified that YTHDF3, but not YTHDF1 or YTHDF2, could bind to the 3′UTR of MCU mRNA that underwent m6A modification and promote translation to facilitate MCU protein expression without affecting mRNA expression. As m6A modification is a dynamic process, I further identified ALKBH5 as a demethylase in the process of MCU m6A modification, and knockdown of ALKBH5 resulted in elevated methylation levels and elevated expression of MCU. Hence, in our current study, we propose a novel model in which METTL3 methylates MCU at three m6A residues in the 3′UTR, thereby promoting the association of RNA-binding protein YTHDF3 with MCU mRNA and promoting the translation of MCU protein. Supporting our findings, one study reported that m6A modification could alter RNA conformation to influence interaction with RNA-binding protein (Liu et al., 2017).

In conclusion, we established a model of HCMV-induced apoptosis in vascular endothelial cells and confirmed that HCMV promoted increased translation of MCU through METTL3 and YTHDF3-dependent manner, thereby promoting apoptosis. Conversely, vitamin D3 can downregulate the METTL3 by inhibiting the activation of AMPK, thereby inhibiting the m6A modification of MCU and cell apoptosis. Our findings extended the understanding of m6A driven machinery in virus-induced vascular endothelium damage and highlighted the significance of vitamin D3 in the intervention of HCMV-induced atherosclerosis.

## Data Availability Statement

The original contributions presented in the study are included in the article/**Supplementary Material**, further inquiries can be directed to the corresponding authors.

## Author Contributions

WZ and HZ designed and performed experiments, analyzed the data, and wrote the manuscript. HZ and SW guided the research and critically revised the manuscript. All authors have read and approved the final manuscript.

## Funding

This work was supported by the grants from the Natural Science Foundation of Hunan province (no. 2020JJ8031) and the cooperation project of FAAS (DWHZ2021-02).

## Conflict of Interest

The authors declare that the research was conducted in the absence of any commercial or financial relationships that could be construed as a potential conflict of interest.

## Publisher’s Note

All claims expressed in this article are solely those of the authors and do not necessarily represent those of their affiliated organizations, or those of the publisher, the editors and the reviewers. Any product that may be evaluated in this article, or claim that may be made by its manufacturer, is not guaranteed or endorsed by the publisher.

## Supplementary Material

Supplementary Figure 1 **(A–C)** Western blot analysis for knockdown efficiency of YTHDF1 **(A)**, YTHDF2 **(B)**, and MCU **(C)** in HAECs with siRNA. **(D)** Effect of MCU activity on caspase-3 and caspase-9 activities using Caspase-3/9 fluorescent assay kits. **(E)** Knockdown of METTL14 in HAECs does not affect MCU protein expression. **(F)** Overexpression of METTL14 in HAECs does not affect MCU protein expression. **(G)** Sequence display of MCU 3′-UTR with mutated m6A sites. All data are expressed as the mean ± SD of triplicate experiments. Values of *p* were calculated with student’s *t*-test, ^**^*p* < 0.01 and ^***^*p* < 0.001. ns: no significant difference.

## Abbreviations

HCMV: Human cytomegalovirus
VDR: Vitamin D receptor
m6A: N^6^-methyladenosine
MCU: Mitochondrial calcium uniporter
PTMs: Post-translational modifications
HAECs: Primary human aortic endothelial cells
RPE-1: Retinal pigment epithelial cells
Bcl-2: B-cell lymphoma-2
Bax: Bcl2-Associated X
GRP78: Glucose-regulated protein 78
CHOP: C/EBP homologous protein
AMPK: Adenosine 5′-monophosphate (AMP)-activated protein kinase
PGC-1α: Peroxisome proliferator-activated receptor-γcoactivator-1α
TFAM: Transcription factor A, mitochondrial
NRF-1: Nuclear respiratory factor 1
ATP5G1: ATP synthase, H+ transporting, mitochondrial F0 complex, subunit C1(subunit 9)
Cox5a: Cytochrome *c* oxidase subunit 5A
MFN1: Mitofusin 1
MFN2: Mitofusin 2
Drp 1: Dynamin related protein 1
FIS 1: Fission, mitochondrial 1
MTFP1: Mitochondrial fission process 1
METTL3: Methyltransferase 3, N6-adenosine-methyltransferase complex catalytic subunit
METTL14: Methyltransferase 14, N6-adenosine-methyltransferase subunit
WTAP: WT1 associated protein
ALKBH5: AlkB homolog 5, RNA demethylase
FTO: Fat mass and obesity-associated protein
YTHDC1: YTH domain containing 1
YTHDF1: YTH N6-methyladenosine RNA-binding protein 1
YTHDF2: YTH N6-methyladenosine RNA-binding protein 2
YTHDF3: YTH N6-methyladenosine RNA-binding protein 3
