# Supplementary figures and images for: Vitamin D3 Suppresses Human Cytomegalovirus-Induced Vascular Endothelial Apoptosis via Rectification of Paradoxical m6A Modification of Mitochondrial Calcium Uniporter mRNA, Which Is Regulated by METTL3 and YTHDF3
Source: Front Microbiol. 2022 Mar 11;13:861734. doi: 10.3389/fmicb.2022.861734 (PMC8963461; doi:10.3389/fmicb.2022.861734)

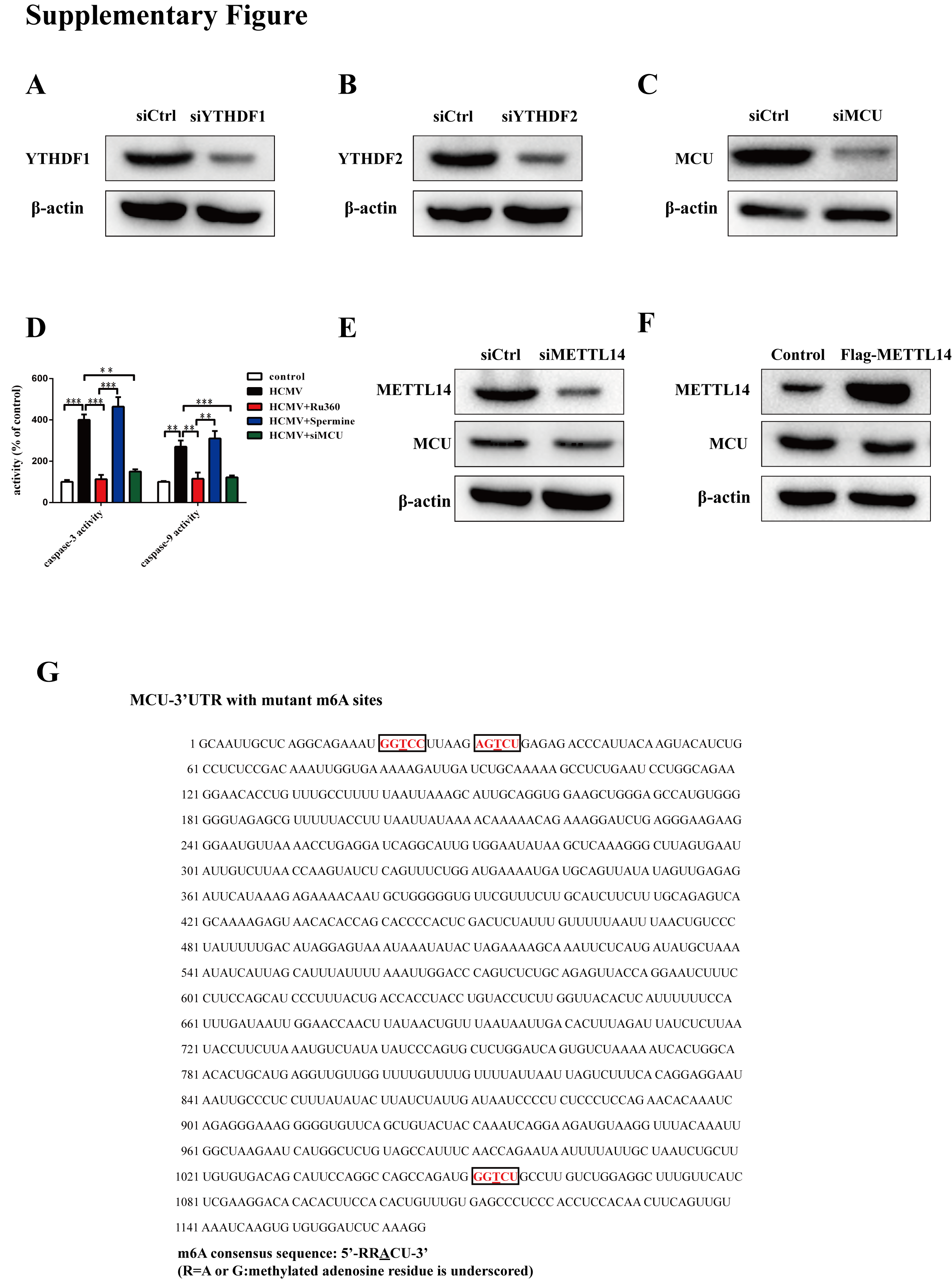

Supplement: Supplementary Figure 1 — (A–C) Western blot analysis for knockdown efficiency of YTHDF1 (A), YTHDF2 (B), and MCU (C) in HAECs with siRNA. (D) Effect of MCU activity on caspase-3 and caspase-9 activities using Caspase-3/9 fluorescent assay kits. (E) Knockdown of METTL14 in HAECs does not affect MCU protein expression. (F) Overexpression of METTL14 in HAECs does not affect MCU protein expression. (G) Sequence display of MCU 3′-UTR with mutated m6A sites. All data are expressed as the mean ± SD of triplicate experiments. Values of p were calculated with student’s t-test, **p < 0.01 and ***p < 0.001. ns: no significant difference. [file Image_1.TIF]
